# Mucopolysaccharidosis Type IVA: Extracellular Matrix Biomarkers in Cardiovascular Disease

**DOI:** 10.3389/fcvm.2022.829111

**Published:** 2022-05-10

**Authors:** Brittany Montavon, Linda E. Winter, Qi Gan, Amirhossein Arasteh, Adriana M. Montaño

**Affiliations:** ^1^Department of Pediatrics, School of Medicine, Saint Louis University, St. Louis, MO, United States; ^2^School of Medicine, Saint Louis University, St. Louis, MO, United States; ^3^Department of Biochemistry and Molecular Biology, School of Medicine, Saint Louis University, St. Louis, MO, United States

**Keywords:** Morquio A, MPS IVA, cardiovascular disease, biomarkers, cathepsin S, elastin

## Abstract

Cardiovascular disease (CVD) in Mucopolysaccharidosis Type IVA (Morquio A), signified by valvular disease and cardiac hypertrophy, is the second leading cause of death and remains untouched by current therapies. Enzyme replacement therapy (ERT) is the gold-standard treatment for MPS disorders including Morquio A. Early administration of ERT improves outcomes of patients from childhood to adulthood while posing new challenges including prognosis of CVD and ERT’s negligible effect on cardiovascular health. Thus, having accurate biomarkers for CVD could be critical. Here we show that cathepsin S (CTSS) and elastin (ELN) can be used as biomarkers of extracellular matrix remodeling in Morquio A disease. We found in a cohort of 54 treatment naïve Morquio A patients and 74 normal controls that CTSS shows promising attributes as a biomarker in young Morquio A children. On the other hand, ELN shows promising attributes as a biomarker in adolescent and adult Morquio A. Plasma/urine keratan sulfate (KS), and urinary glycosaminoglycan (GAG) levels were significantly higher in Morquio A patients (*p* < 0.001) which decreased with age of patients. CTSS levels did not correlate with patients’ phenotypic severity but differed significantly between patients (median range 5.45–8.52 ng/mL) and normal controls (median range 9.61–15.9 ng/mL; *p* < 0.001). We also studied α -2-macroglobulin (A2M), C-reactive protein (CRP), and circulating vascular cell adhesion molecule-1 (sVCAM-1) in a subset of samples to understand the relation between ECM biomarkers and the severity of CVD in Morquio A patients. Our experiments revealed that CRP and sVCAM-1 levels were lower in Morquio A patients compared to normal controls. We also observed a strong inverse correlation between urine/plasma KS and CRP (*p* = 0.013 and *p* = 0.022, respectively) in Morquio A patients as well as a moderate correlation between sVCAM-1 and CTSS in Morquio A patients at all ages (*p* = 0.03). As the first study to date investigating CTSS and ELN levels in Morquio A patients and in the normal population, our results establish a starting point for more elaborate studies in larger populations to understand how CTSS and ELN levels correlate with Morquio A severity.

## Introduction

Mucopolysaccharidosis IVA (MPS IVA; Morquio A syndrome) is an inherited, autosomal recessive disease caused by a deficiency of N-acetylgalactosamine-6-sulfate sulfatase (GALNS), which results in excessive lysosomal storage of keratan sulfate (KS) and chondroitin 6-sulfate (C6S) in many tissues and organs. This accumulation leads to chronic and progressive clinical findings including but not limited to systemic skeletal dysplasia, joint abnormalities, short stature, hearing and vision loss, coarse facial features, valvular heart disease, and pulmonary compromise (1–3). The extent of GALNS deficiency is highly variable thus resulting in a heterogenous clinical presentation (2, 3). Early diagnosis of Morquio A is important to employ disease modifying treatments to slow progression as no cure exists (4).

Currently available therapies for Morquio A include enzyme replacement therapy (ERT) with recombinant human GALNS enzyme, hematopoietic stem cell therapy (HSCT), and orthopedic surgeries. ERT is the most widely used therapeutic option in many lysosomal storage disorders including Morquio A. The enzyme, Elosulfase alfa, works within lysosomes to catabolize the glycosaminoglycans (GAGs) that have accumulated within. ERT has shown clinical improvement in somatic manifestations, activities of daily living, and quality of life (5–9). It has a low mortality risk with no limitations on use, however, the enzymes have a short half-life with rapid clearance from circulation resulting in poor delivery to bone and avascular tissues such as cartilage and cornea even when started at a young age. Other limitations include the need for weekly treatments, hypersensitivity reactions, and high cost (1). Another treatment option is HSCT which is a one-time permanent treatment allowing continuous production of the enzyme. HSCT has shown improvement in pulmonary function, activities of daily living, bone mineral density, cardiovascular involvement, and reduction in number of surgical interventions (10–12). However, it has many health and age limitations, requires a matched donor, carries a high risk of mortality and complications, and has limited effect on bone and cartilage (10, 11, 13). Morquio A patients often develop severe skeletal dysplasia requiring multiple orthopedic surgical interventions in the upper cervical spine and lower extremities as well as tracheal obstruction surgery. ERT and HSCT have limited to no impact on skeletal dysplasia (1, 3, 14).

Although these therapeutic treatments have extended life expectancy for Morquio A patients by attenuating disease progression, they have shown only a limited effect on heart disease which is the second leading cause of death in this population (1, 3, 15, 16). Cardiovascular disease (CVD) in these patients emerges silently and contributes significantly to early mortality (17). Cardiovascular involvement has been reported in Morquio patients as early as 3.6 months of age with subsequent progression of the disease (18). A wide range of cardiac findings have been associated with Morquio A including mitral and/or aortic insufficiency, aortic stenosis, tricuspid regurgitation, thickened interventricular septum, hypertrophic cardiomyopathy, and aortic root dilatation (15, 17–20). Furthermore, post-mortem histopathology on a 20-year-old patient has shown valvular infiltration by foam cells and macrophages along with GAG deposition (particularly C6S), and fragmented, attenuated elastic fibers in the aortic intima and media. These findings imply aortic intima thickening, atherosclerotic plaques, and aortic valve hypertrophy (21). A recent study of 12 Morquio A patients has revealed increased carotid intima media thickness and carotid hyperelasticity. These findings may be explained by alteration of arterial elasticity by GAG bearing proteoglycans (16). Limited therapeutic treatments exist for Morquio A that can address the CVD. ERT has poor delivery of the enzyme to cardiac valves and the aorta because of its short half-life in circulation, effect on cartilaginous tissues, and low density of mannose 6 phosphate receptors (1, 22). Another explanation of the slight therapeutic efficacy of ERT in heart has been attributed historically to the “avascular” nature of heart valves, which remains to be a debatable topic that needs further investigation in Morquio A patients (23). ERT studies with short term echocardiographic follow up have shown only a negligible therapeutic effect on cardiovascular findings (1, 15, 16, 20, 22, 24). The incidence of cardiac surgery in Morquio A patients is low (2%) compared to other surgeries. However, the most common cardiac surgery in adult Morquio A patients is aortic valve replacement (3, 25–27). Thus, development of a therapy to ameliorate CVD could be lifesaving in Morquio A patients.

Attenuated and fragmented elastin fibrils have been found not only in the aortic intima of a Morquio A patient (21) but also in patients and animal models of other MPS types, such as MPS I, VI, VII (28–30), and IVA (unpublished). Elastin fragmentation is the result of proteolysis by cathepsins and the matrix metalloproteinases secreted by GAG-activated macrophages (28, 31). It results in acquired CVD including atherosclerosis, calcification, and aneurysms. Elastic fiber degradation allows infiltration of lipids and immune cells into the aortic wall for plaque formation and rupture. The products from degradation further enhance atherogenesis. Calcification in the arterial medial wall and aneurysmal formation have also been linked to elastic fiber degradation and fragmentation. Fragmented elastic fibers may be more susceptible to protease digestion leading to aneurysmal dilation and stimulation and signaling of cytokine and inflammatory cell infiltration (32).

Elastin degradation leads to an irreversible and difficult to halt cascade of arterial remodeling, and cytokine and inflammatory signaling pathway activation. Damaged and degraded elastic fibers are generally not repaired due to the limited timeframe of elastin synthesis (32). Elastin is a highly abundant hydrophobic extracellular matrix (ECM) protein within the arterial media, and it is arranged in tightly coiled laminae. It is responsible for the resilience and elasticity of large arteries allowing them to withstand blood pressure oscillations throughout the cardiac cycle (32, 33). Elastic fibers can be easily degraded by specific proteases called elastases. One of these elastases, cathepsin S, is a lysosomal cysteine protease highly expressed in GAG-bearing macrophages (32, 34). Secreted cathepsin S at the basal membrane of blood vessels cleaves several ECM proteins including laminin, collagen, and preferentially elastin which generates bioactive elastin peptides (35). This potent elastolytic and collagenolytic activity has been linked to vascular inflammation and calcification resulting in the acquired CVD atherosclerosis and aneurysmal formation (32, 34, 36). Although protein levels and/or gene expression of elastin and cathepsin S have not been explored in Morquio A patients, we have observed dysregulation with age (unpublished) in Morquio A mice. Thus elastin and cathepsin S may be potential biomarkers to predict the severity of heart disease and phenotype of this patient population.

## Materials and Methods

### Study Populations

Plasma and urine samples of Morquio A patients were obtained from a de-identified repository located at Saint Louis University. The Institutional Review Board (IRB) at Saint Louis University determined that our human subjects research was exempt from a formal IRB submission due to the lack of patient identifiers or protected health information (PHI). Morquio A patients from the de-identified repository were previously diagnosed based on clinical phenotype, elevated KS in blood, elevated GAG levels in urine, and by having reduced GALNS enzyme activity below 5% of the normal level in leukocytes and/or by having two or more pathogenic mutations in the GALNS gene. Morquio A patients were classified by phenotype as mild or severe based on gene mutation effects and by patient height (severe phenotype with a lower 90th centile height isopleth in Morquio A growth charts) (2). Mutation effects were determined by *in vitro* assays (37–39) and by using the PolyPhen-2 program (40). Age-matched normal controls were obtained from a de-identified repository from Saint Louis University and Cardinal Glennon Children’s Hospital.

### Measurement of Glycosaminoglycans

Concentrations of urinary and plasma GAGs and KS were measured by tandem mass spectroscopy as described elsewhere (16). Briefly, the chromatographic system consists of an HP1100 system (Agilent Technologies) and a Hypercarb column (Thermo Electron). The API-4000 mass spectrometer (Applied Biosystems) was equipped with a turbo ionspray ion source. Disaccharides of keratan, heparan, dermatan sulfate were used as standards [Galß1,4GlcNAc(6S), ΔDiHS-0S, ΔDiHS-NS, ΔDi-4S, ΔDi-6S (Seikagaku). Chondrosine was used as internal standard (Glycosyn). Urine or plasma was centrifuged, and the supernatants were digested overnight with 1 mU of chondroitinase b, 1mU heparitinase, and 1mU keratanase II (Seikagaku). Recovered samples were analyzed by the LC-MS/MS system and normalized by creatinine concentration in urine. Total GAGs refer to total glycosaminoglycans or the additive sum of all the GAGs measured (HS, DS and KS). Disaccharide GAG concentrations were calculated by Analyst 1.5.1 software (AB SCIEX). Each sample was measured in triplicate with three injections for each sample.

### Investigational Biomarkers

Plasma concentrations of cathepsin S were measured in triplicate by enzyme-linked immunosorbent assay (ELISA) (R&D systems) after diluting the samples 1:100. Plasma concentrations of elastin were determined in duplicate by ELISA (Abcam) after diluting the samples 1:4 following the manufacturer’s instructions. C-reactive protein (CRP), α-2-macroglobulin (A2M), and circulating vascular cell adhesion molecule-1 (sVCAM-1) were detected in triplicates from plasma samples by LUMINEX technology, using a Millipore Milliplex kit according to the manufacturer’s instructions.

### Statistical Analysis

Data was expressed as the median values and interquartile range. *P*-values were determined by Kruskal-Wallis test, Mann–Whitney *U*-test, Chi-square test, or One-Way ANOVA where appropriate. Multiple comparisons were corrected using Šidák’*s*-test. Pearson correlations coefficients were used to assess linear correlations between age, KS, GAG, cathepsin S, elastin, CRP, A2M, and sVCAM-1 levels. Receiver operating characteristic (ROC) analyses of plasma cathepsin S and elastin levels were performed to examine the sensitivity and specificity of plasma cathepsin S and elastin levels as biomarkers for CVD in all age groups. *P*-values <0.05 were considered statistically significant. Statistical analyses were performed on Prism 9.0 (GraphPad, San Diego, CA, United States).

## Results

### Subject Characteristics and Comparisons of Extracellular Matrix Biomarkers

Fifty-four Morquio A patients and 74 age-matched controls were included in this study. Morquio A patients were identified as mild (*n* = 19) or severe (*n* = 35) phenotype (Supplementary Table 1). Forty-three different pathogenic mutations were identified, 95% causing mild or severe phenotype. The median age of Morquio A patients is similar amongst severity and consistent with pediatric age range (median [IQR] mild, 9 [7–14] years and severe, 8 [5.5–13] years) (Table 1). Present height and weight were significantly different among Morquio A patient severity (*p* < 0.001), which has been previously described (2).

**TABLE 1:** Demographic and biomarker comparisons of Morquio A patients by severity and normal controls.

	Mild	Severe	Normal adults	Normal pediatrics	All 4 groups	Morquio A Pts
	(*n* = 19)	(*n* = 35)	(*n* = 22)	(*n* = 52)		
Demographics	Median(IQR); n	Median(IQR); n	Median(IQR); n	Median(IQR); n	*P*-value^a^	*P*-value^b^
Age (years)	9.0 (7.0 – 14.0); 19	8.0 (5.5 – 13.0); 35	40 (33.75 – 46.0); 22	13.0 (8.0 – 15.0); 52	<0.001	0.29
Age group, n(%)					<0.001	0.93
<18 years	15 (78.9)	28 (80.0)	0	48 (92.3)		
>=18 years	4 (21.1)	7 (20.0)	22 (100)	4 (7.7)		
Gender, n(%)					0.34	0.06
Male	13 (72.2)	15 (45.5)	12 (54.5)	28 (54.9)		
Female	5 (27.8)	18 (54.5)	10 (45.5)	23 (45.1)		
Birth Weight(g)	3570.0 (3327.0 – 4029.0); 13	3762.0 (3343.0 – 4340.0); 18	–	–	–	0.34
Birth Height(cm)	53.0 (51.0 – 54.6); 11	53.3 (50.9- 55.9); 17	–	–	–	0.67
Present Weight(kg)	23.78 (20.5 – 37.4); 16	15.0 (14.0–16.8); 27	–	–	–	<0.001
Present Height(cm)	118.5 (112.9 – 133.2); 16	94.0 (90.0 – 101.6); 27	–	–	–	<0.001
**Biomarkers**						
Cathepsin S (ng/mL)	6.91 (4.83– 9.52); 19	6.0 (4.8– 9.32); 35	9.94 (8.56 – 12.45); 22	11.4 (8.75 – 15.83); 52	<0.001	0.58
Elastin (ng/mL)	3.48 (2.45 – 4.8); 19	3.25 (2.24 – 4.51); 35	3.26 (2.38 – 4.29); 22	3.34 (2.26 – 4.85); 52	0.9	0.58
Plasma KS (ng/mL)	577.0 (370.8 – 1114.3); 17	681.3 (380.0 – 2807.0); 29	159.2 (112.3 – 201.5); 20	56.58 (33.24 – 87.41); 50	<0.001	0.56
Urine GAGs (mg/gCr)	167.5 (87.55 – 248.5); 16	186.2 (92.93 – 330.8); 28	6.45 (4.45 – 12.03); 8		(<0.001)^#^	0.57
Urine KS (mg/gCr)	2.17 (1.27 – 6.73); 16	7.24 (3.04 – 13.3); 26	0.08 (0.05 – 0.11); 9		(<0.001)^#^	0.02
Plasma GAGs (ng/mL)				98.76 (63.09 - 861.6); 48		

*IQR, Interquartile Range; GAGs, Total glycosaminoglycans.*

*^a^p-value for Normal, Mild, and Severe; Kruskal-Wallis test for continuous variables and Chi-square tests for categorical variable.*

*^b^p-value for Mild versus Severe only; Mann-Whitney U-test for continuous variables and Chi-square test for categorical variables.*

*^#^p-value for Normal Adult, Mild, and Severe; Kruskal–Wallis test.*

Cathepsin S levels differed significantly between patients and normal controls (*p* < 0.001). In contrast, there was no significant difference of elastin levels between Morquio A patients and controls. Plasma KS, urine GAGs, and urine KS levels were significantly higher in Morquio A patients (*p* < 0.001), consistent with previous literature (41–44), however, displayed no significant difference amongst severity except for urine KS levels (median [IQR] mild, 2.17 [1.27–6.73] mg/gCr and severe, 7.24 [3.04–13.3] mg/gCr; *p* = 0.02).

### Age Group Dependent Analysis of Extracellular Matrix Biomarkers

To understand if there was an age dependent effect of cathepsin S and elastin, Morquio A patients and normal controls were stratified by age groups. Cathepsin S levels were similar amongst Morquio A patient age groups (median range 5.45–8.52 ng/mL; Table 2), however, differed from normal controls (*p* < 0.001) with higher median levels (median range 9.61–15.9 ng/mL). Normal controls 0 to 5 years old had the highest level (median [IQR] 15.9 [12.3–19.98] ng/mL). Elastin levels differed amongst Morquio A patients and normal controls (*p* = 0.02) with highest median levels in Morquio A patients >40 years old (median [IQR] 5.19 [4.19– 5.87] ng/mL) and normal controls >5 to 10 years old (median [IQR] 4.99 [3.42 – 8.29] ng/mL). As expected, and previously described, urinary GAGs and KS levels as well as plasma KS levels were the highest in Morquio A childhood (41). Urinary GAGs and KS differed between all age groups (*p* < 0.001); however, we did not obtain urine samples in the normal pediatric population which limits the available data for comparison. Plasma KS was significantly different between all age groups (*p* < 0.001). Plasma KS levels of Morquio A 0–5 years old group was twenty-two times higher than that of the normal control group of the same age. Similarly, plasma KS levels of Morquio A >5–10 years old were ten times higher than the plasma KS levels of normal controls of the same age. Morquio A group >10–15 years old showed 6.5 times higher plasma KS levels compared to the normal age-matched controls. Plasma GAG data was only available for normal age groups 0–20 years with concentrations the highest in early childhood, specifically 0 to 5 years old (median [IQR] 1139.0 [86.97 – 1374.0] ng/mL). These findings confirm that plasma GAGs are elevated in childhood and decreased with age (45).

**TABLE 2:** Biomarker comparisons of Morquio A and normal control age groups.

	Morquio A						Normal						All age
	0–5 yrs	>5–10 yrs	>10–15 yrs	>15–20 yrs	>20—40 yrs	>40 yrs	0–5 yrs	>5–10 yrs	>10–15 yrs	>15–20 yrs	>20–40 yrs	>40 yrs	Groups

	**(*n* = 10)**	**(*n* = 23)**	**(*n* = 10)**	**(*n* = 2)**	**(*n* = 5)**	**(*n* = 4)**	**(*n* = 9)**	**(*n* = 9)**	**(*n* = 23)**	**(*n* = 11)**	**(*n* = 17)**	**(*n* = 9)**	
**Biomarkers**	**Median(IQR); n**	**Median(IQR); n**	**Median(IQR); n**	**Median(IQR); n**	**Median(IQR); n**	**Median(IQR); n**	**Median(IQR); n**	**Median(IQR); n**	**Median(IQR); n**	**Median(IQR); n**	**Median(IQR); n**	**Median(IQR); n**	***P*-value^a^**
Age (yrs)	3.75 (1.98–4.33)	7.0 (6.3–8.5)	11 (10.38–13.0)	18.6 (18.2–19.0)	24.0 (23.65–32.50)	46.9 (45.83–54.65)	3.0 (3.0–5.0)	8.0 (7.0–10.0)	14.0 (13.0–14.0)	17.0 (17.0–18.0)	37.0 (32.0–39.5)	46.0 (43.8–50.5)	<0.001
Cathepsin S (ng/mL)	6.71 (5.03–9.55); 10	7.01 (4.76–9.42); 23	5.45 (3.72–8.57); 10	8.52 (5.74–11.29); 2	6.84 (4.33–10.01); 5	8.03 (5.29–10.96); 4	15.9 (12.3–19.98); 9	9.05 (6.65–9.72); 9	11.53 (8.72–16.28); 23	9.91 (7.98–15.60); 11	9.61 (7.07–11.89); 13	11.27 (9.36–15.31): 9	<0.001
Elastin (ng/mL)	3.53 (2.41–4.26); 10	3.64 (2.24–4.65); 23	3.16 (1.67–3.90); 10	3.88 (3.1–4.67); 2	2.64 (1.46–3.48); 5	5.19 (4.19–5.87); 4	2.74 (2.36–3.19); 9	4.99 (3.42–8.29); 9	3.49 (1.96–4.81); 23	3.07 (1.56–4.67); 11	2.93 (2.03–3.36); 13	4.58 (3.51–4.84); 9	0.02
Urine GAGs (mg/gCr)	384.1 (201.8–532.5); 6	191.0 (144.9–322.0); 20	160.0 (84.67–281.0); 9	156.3 (91.58–221.0); 2	49.95 (30.70–164.6); 4	97.0 (87.1–103.0); 3	–		–	–	6.45 (4.68–23.0); 4	7.05 (3.65–12.03); 4	<0.001
Urine KS (mg/gCr)	8.35 (6.18–10.55); 6	8.37 (1.74–13.6); 19	3.14 (1.67–8.58);8	3.38 (3.04–3,71); 2	1.9 (0.76–2.8); 4	1.72 (0.97–2.17); 3	–		–	–	0.07 (0.04–0.1); 4	0.1 (0.06–73.91); 5	<0.001
Plasma KS (ng/mL)	1733.0 (524.0–4807.0); 8	629.0 (531.0–1388.0); 19	381.0 (272.0–2959); 9	2212 (299.0–4125.0); 2	296.5 (115.2–2228.0); 4	180.4 (115.5–1189.0); 4	78.21 (52.61–120.1); 9	62.71 (25.83–87.13); 7	59.14 (39.14–91.84); 23	25.95 (12.45–41.58); 11	155.5 (90.78–250.0); 12	159.2 (137.5–188.4); 8	<0.001
Plasma GAGs (ng/mL)	–	–	–	–	–	–	1139.0 (86.97–1374.0); 9	104.2 (61.89–449.1); 7	123.0 (67.95–858.9); 21	64.29 (59.82–67.52); 11		–	0.05

*IQR, Interquartile Range; GAGs, Total glycosaminoglycans;*

*^a^p-value; Kruskal–Wallis test.*

### Identical Age Group Analysis of Extracellular Matrix Biomarkers

We observed differences of cathepsin S and elastin levels when the patients were grouped by age. Cathepsin S levels differed in early childhood (0–5 years old) and early adolescence (>10–15 years old, *p* < 0.001; Table 3) between Morquio A patients and normal controls. In addition, elastin levels were significantly different amongst >5–10 years old (*p* = 0.02) Morquio A patients and normal controls. Urinary GAG and KS levels were significantly different between Morquio A and normal controls >20–40 years old (*p* = 0.03). Plasma KS levels differed significantly amongst all age groups from 0 to 20 years old.

**TABLE 3:** Biomarker comparisons between age groups of Morquio A and normal controls.

Biomarkers	Morquio A vs. Normal controls					
	0–5 yrs	>5–10 yrs	>10–15 yrs	>15–20 yrs	>20–40 yrs	>40 yrs
	
	*P*-value	*P*-value	*P*-value	*P*-value	*P*-value	*P*-value
Age (yrs)	0.28	0.12	<0.001	0.05	0.02	0.69
Cat S (ng/mL)	<0.001	0.24	<0.001	0.41	0.17	0.11
Elastin (ng/mL)	0.28	0.02	0.34	0.53	0.7	0.26
Urine GAGs (mg/gCr)	–	–	–	–	0.03	0.06
Urine KS (mg/gCr)	–	–	–	–	0.03	0.25
Plasma KS (ng/mL)	<0.001	<0.001	<0.001	0.03	0.38	0.57

*P-value; Mann–Whitney test.*

### Association Between Extracellular Matrix Biomarkers and Glycosaminoglycans

**FIGURE 1:**
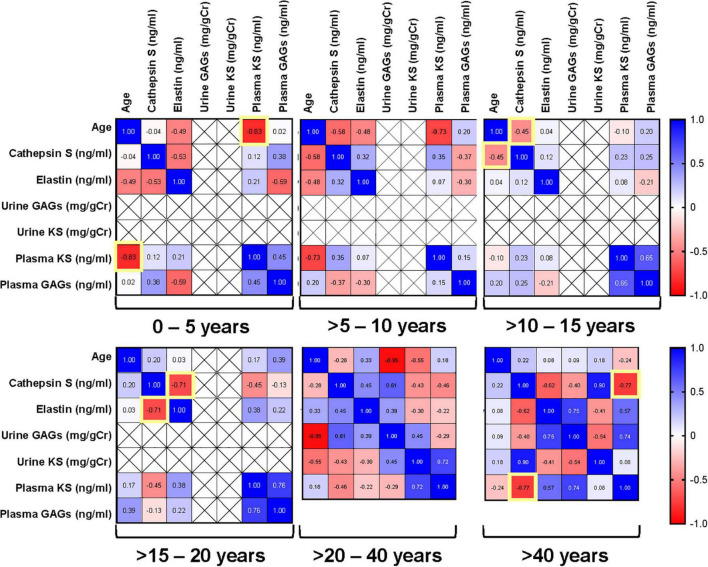
Pairwise Pearson correlation matrix of age, keratan sulfate (KS), glycosaminoglycans (GAGs), cathepsin S and elastin in normal samples stratified by age groups. Blue indicates positive correlation, and red indicates negative correlation. Darker colors are associated with stronger correlation coefficients. Yellow boxes indicate significant correlations.

To determine the association of GAGs, elastin, cathepsin S, and age in normal population, we measured their correlation with each other. A strong correlation existed among all age groups for plasma GAGs and KS (*p* < 0.001) and a moderate correlation was identified for plasma GAGs and cathepsin S (*p* = 0.02) (not shown). A strong inverse correlation was present between various biomarkers and age groups including cathepsin S and elastin in >15–20 years old (*p* = 0.015), plasma KS and cathepsin S in >40 years old (*p* = 0.026). In addition, there was a strong inverse correlation between age and plasma KS in 0–5 years old (*p* < 0.005), and a moderate inverse correlation between age and cathepsin S for >10–15 years old (*p* = 0.031) (Figure 1).

**FIGURE 2:**
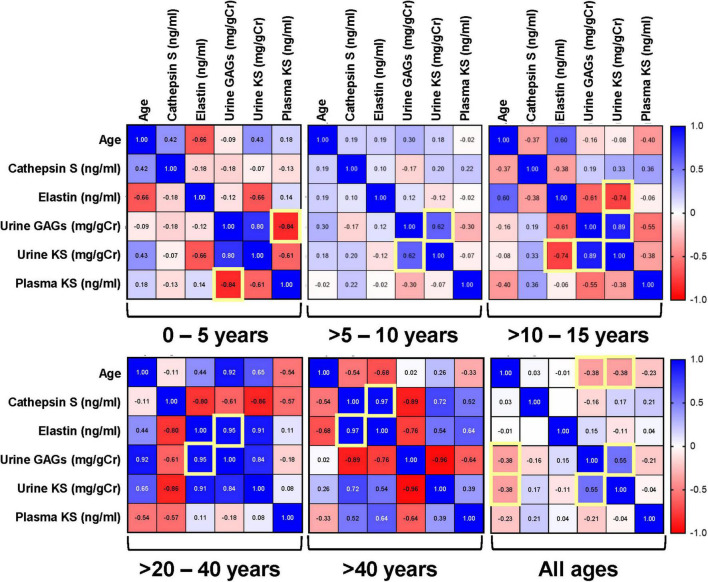
Pairwise Pearson correlation matrix of age, keratan sulfate (KS), glycosaminoglycans (GAGs), cathepsin S and elastin in Morquio A samples stratified by age groups. Blue indicates positive correlation, and red indicates negative correlation. Darker colors are associated with stronger correlation coefficients. Yellow boxes indicate significant correlations. The term “all ages” refers to the compound group of different ages that are part of this study.

To understand the relationship of cathepsin S, elastin, and GAG production in Morquio A patients, we determined correlation between them at different age groups. There was a strong direct correlation between urine GAGs and urine KS in all age groups of Morquio A patients (*p* < 0.001; Figure 2), consistent with previous studies (46, 47). A strong correlation was also present between urine GAGs and elastin in patients >20–40 years old (*p* = 0.046), and elastin and cathepsin S in >40 years old (*p* = 0.029). A strong inverse correlation between urine KS and elastin was displayed in >10–15 years old (*p* = 0.035).

### Discrimination of Cathepsin S and Elastin as Extracellular Matrix Biomarkers for Morquio A Disease

**FIGURE 3:**
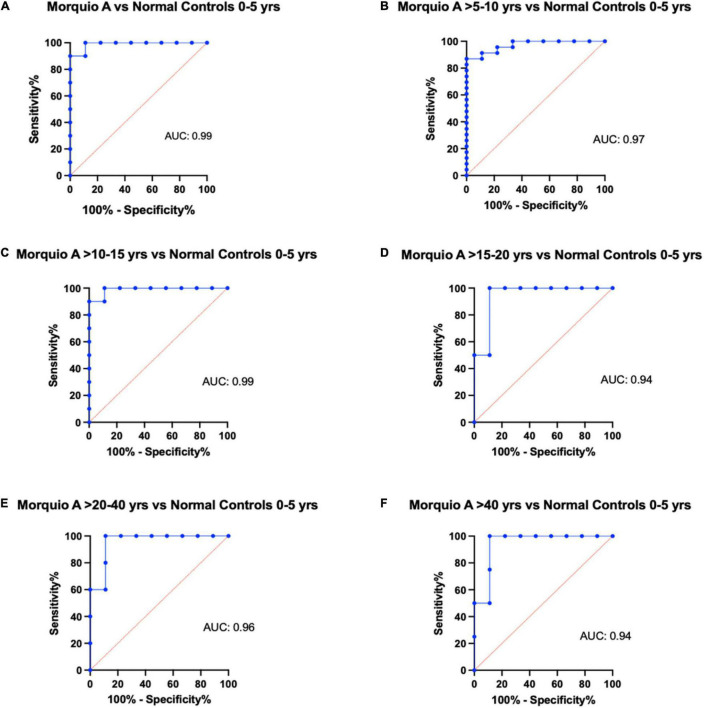
Representative figures of receiver operating characteristic (ROC) curves for cathepsin S comparing different age groups. Comparison of the diagnostic ability of Cathepsin S in the Morquio A cohort and the normal cohort stratified by age. Comparison of normal controls 0 to 5 years of age with: **(A)** Morquio A patients 0 to 5 years of age, **(B)** Morquio A patients >5 to 10 years of age, **(C)** Morquio A patients >10 to 15 years of age, **(D)** Morquio A patients >15–20 years of age, **(E)** Morquio A patients >20 to 40 years of age, and **(F)** Morquio A patients >40 years of age. AUC, Area under the curve.

**FIGURE 4:**
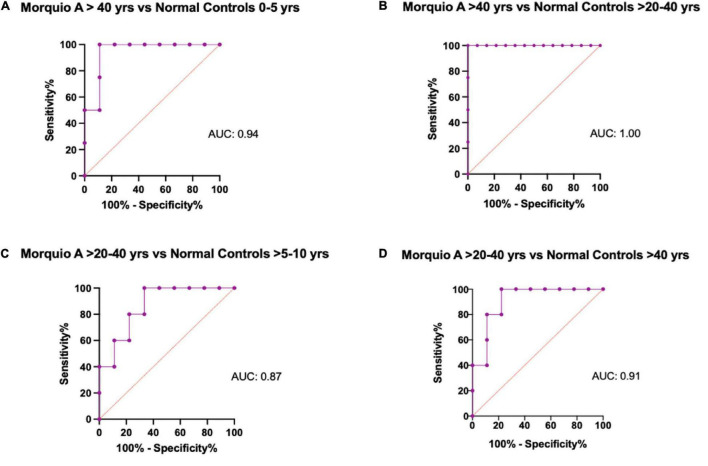
Representative figures of receiver operating characteristic (ROC) curves for Elastin comparing different age groups. Comparison of the diagnostic ability of Elastin in the Morquio A cohort and the normal cohort stratified by age. **(A)** Morquio A patients >40 years of age vs. Normal controls 0 to 5 years of age, **(B)** Morquio A patients >40 years of age vs. Normal controls >20 to 40 years of age, **(C)** Morquio A patients >20 to 40 years of age vs. Normal controls 5 to 10 years of age, **(D)** Morquio A patients >20 to 40 years of age vs. Normal controls >40 years of age. AUC, Area under the curve.

To evaluate the diagnostic power of elastin and cathepsin S as potential biomarkers to discriminate between Morquio A and normal controls at different age groups, we performed receiver operating characteristic (ROC) curves. The ROC curves help to evaluate the discriminating ability of a biomarker by providing information between its sensitivity (true positive rate) and specificity (1- false positive rate). Values of the area under the ROC curve close to 1 indicate that the biomarker has high diagnostic accuracy, while a value of 0.5 has no predictive value (48, 49). ROC curve profiles suggest that cathepsin S levels can be used to discriminate Morquio A patients of any age from controls 0–5 years old (AUC >0.9 (*p* = 0.0003); Figures 3A–F). Similarly, ROC curve profiles suggest that Cathepsin S levels can be used to discriminate Morquio A children of various ages [0–15 years old] from normal controls of all ages (AUC >0.8). ROC curve profiles reflect that elastin levels can be used to discriminate Morquio A patients (>15 years old) from normal controls (AUC >0.8; Table 4). In addition, elastin levels can be clearly distinguished between adult Morquio A patients >40 years old and adult normal controls >20–40 years old (AUC 1.0; (*p* = 0.003); Figures 4A–D).

**TABLE 4:** Receiver operating characteristic area under the curves for cathepsin S and elastin.

Cathepsin S			Elastin		
Morquio A vs. Normal controls age groups (yrs)	AUC	*p*-value	Morquio A vs. Normal controls age groups (yrs)	AUC	*p*-value
**0–5 vs. 0–5**	**0.99**	**0.000**	0–5 vs. 0–5	0.61	0.40
0–5 vs. >5–10	0.78	**0.040**	0–5 vs. >5–10	0.79	**0.03**
**0–5 vs. >10–15**	**0.85**	**0.002**	0–5 vs. >10–15	0.53	0.78
**0–5 vs. >15–20**	**0.92**	**0.002**	0–5 vs. >15–20	0.51	0.93
**0–5 vs. >20–40**	**0.88**	**0.002**	0–5 vs. >20–40	0.69	0.13
**0–5 vs. >40**	**0.93**	**0.001**	0–5 vs. >40	0.77	**0.05**
**>5–10 vs. 0–5**	**0.97**	**<0.0001**	>5–10 vs. 0–5	0.55	0.63
>5–10 vs. >5–10	0.70	0.070	>5–10 vs. >5–10	0.79	**0.01**
>5–10 vs. >10–15	0.78	**0.001**	>5–10 vs, >10–15	0.51	0.86
**>5–10 vs. >15–20**	**0.88**	**0.001**	>5–10 vs. >15–20	0.52	0.87
**>5–10 vs. >20–40**	**0.81**	**0.001**	>5–10 vs. >20–40	0.66	0.09
**>5–10 vs. >40**	**0.87**	**0.001**	>5–10 vs. >40	0.70	0.07
**>10–15 vs. >0–5**	**0.99**	**0.003**	>10–15 vs. >0–5	0.52	0.87
**>10–15 vs. >5–10**	**0.87**	**0.001**	>10–15 vs. >5–10	0.79	**0.03**
**>10–15 vs. >10–15**	**0.87**	**0.001**	>10–15 vs. >10–15	0.61	0.32
**>10–15 vs. >15–20**	**0.94**	**0.001**	>10–15 vs. >15–20	0.53	0.82
**>10–15 vs. >20–40**	**0.88**	**0.001**	>10–15 vs. >20–40	0.57	0.56
**>10–15 vs. >40**	**0.93**	**0.001**	>10–15 vs. >40	0.72	0.10
**>15–20 vs. >0–5**	**0.94**	**0.050**	>15–20 vs. >0–5	**0.83**	0.16
>15–20 vs. >5–10	0.61	0.630	>15–20 vs. >5–10	0.72	0.34
>15–20 vs. >10–15	0.69	0.360	>15–20 vs. >10–15	0.55	0.80
>15–20 vs. >15–20	**0.80**	0.190	>15–20 vs. >15–20	0.63	0.59
>15–20 vs. >20–40	0.59	0.670	>15–20 vs. >20–40	**0.82**	0.15
>15–20 vs. >40	0.75	0.280	>15–20 vs. >40	0.55	0.81
**>20–40 vs. 0–5**	**0.95**	**0.006**	>20–40 vs. 0–5	0.60	0.54
>20–40 vs. >5–10	0.64	0.380	**>20–40 vs. >5–10**	**0.87**	**0.03**
>20–40 vs. >10–15	0.76	0.060	>20–40 vs. >10–15	0.70	0.16
**>20–40 vs. >15–20**	**0.82**	**0.050**	>20–40 vs. >15–20	0.64	0.39
>20–40 vs. >20–40	0.67	0.240	>20–40 vs. >20–40	0.54	0.78
>20–40 vs. >40	** 0.80**	0.060	**>20–40 vs. >40**	**0.91**	**0.01**
**>40 vs. 0–5**	**0.94**	**0.013**	**>40 vs. 0–5**	**0.94**	**0.01**
>40 vs. >5–10	0.53	0.870	>40 vs. >5–10	0.50	>0.999
>40 vs. >10–15	0.64	0.340	>40 vs. >10–15	0.75	0.12
>40 vs. >15–20	0.70	0.250	>40 vs. >15–20	**0.80**	0.09
>40 vs. >20–40	0.56	0.700	**>40 vs. >20–40**	**1.00**	**0.00**
>40 vs. >40	0.65	0.390	>40 vs. >40	0.72	0.22

*AUC: Area under the curve; Yrs: years. In bold: AUC ≥ 0.8, p ≤ 0.05.*

### Extracellular Matrix Biomarker Comparisons Between Morquio A Patients and Normal Controls

**FIGURE 5:**
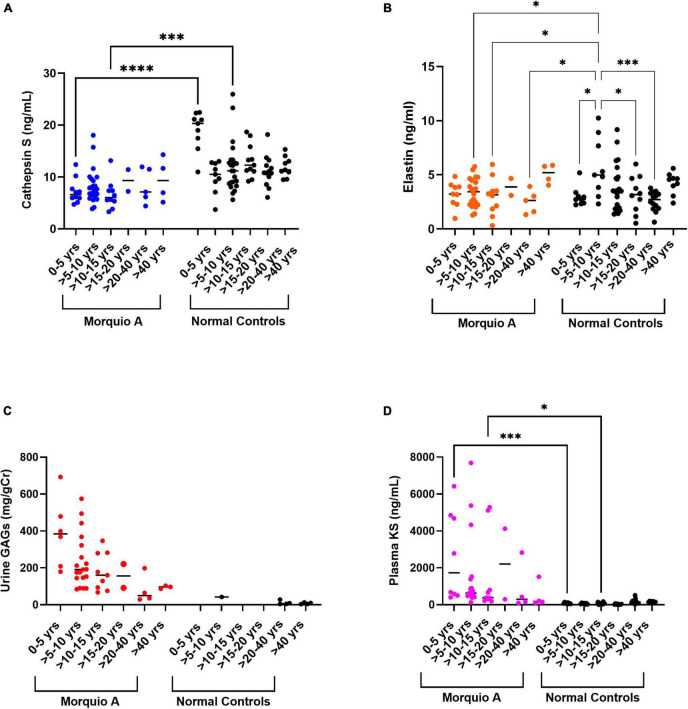
Biomarkers comparisons between Morquio A and normal controls. Comparison of **(A)** Cathepsin S, **(B)** Elastin, **(C)** Urine GAGs or **(D)** Plasma KS, between Morquio A and normal controls stratified by age groups. Significant differences are denoted with asterisks (*p ≤ 0.05, ^***^p ≤ 0.001, and ^****^p ≤ 0.0001). *P*-values were determined by one-way ANOVA followed by ordinary one-way ANOVA multiple comparisons test.

To compare cathepsin S and elastin levels among different age groups, we performed scatter plot analyses in Morquio A patients and normal controls. Cathepsin S levels were significantly different between Morquio A patients and normal controls (*p* < 0.001). Cathepsin S levels in young Morquio A patients 0–5 years old and early adolescence >10–15 years old were lower than those in normal controls of the same age groups (*p* < 0.001 and *p* = 0.005; Figure 5A). Cathepsin S levels in normal controls 0–5 years old were very different than all others. Elastin levels in Morquio A disease seem to increase with age (Figure 5B). On the other hand, elastin levels in normal controls increased with age until 10 years of age and then showed a steady decrease until 40 years of age. Elastin levels in normal controls >5 to 10 years old were significantly higher than elastin levels in i) Morquio A patients in the same age group (*p* = 0.017), ii) Morquio A patients >10–15 years old (*p* = 0.02), and iii) Morquio A patients >20–40 years old (*p* = 0.02).

To confirm previous findings that showed a steady decrease of GAG levels with age (45, 47), we made a comparison of urine GAGs and plasma KS in Morquio A patients and normal controls. Urine GAG levels contrasted between Morquio A patients and normal controls (*p* = 0.0001; Figure 5C). There was a dramatic reduction of urine GAG levels in Morquio A patients from 5 years of age until having values close to the ones of normal controls after 20 years of age. Plasma KS levels differed between Morquio A patients and normal controls (*p* < 0.0001; Figure 5D). Significant difference of plasma KS levels was displayed between 0–5 years old and >10–15 years old age groups (*p* < 0.001 and *p* = 0.03) of Morquio A patients and normal controls.

### Comparison of Cardiovascular Disease Biomarkers and Extracellular Matrix Biomarkers

**FIGURE 6:**
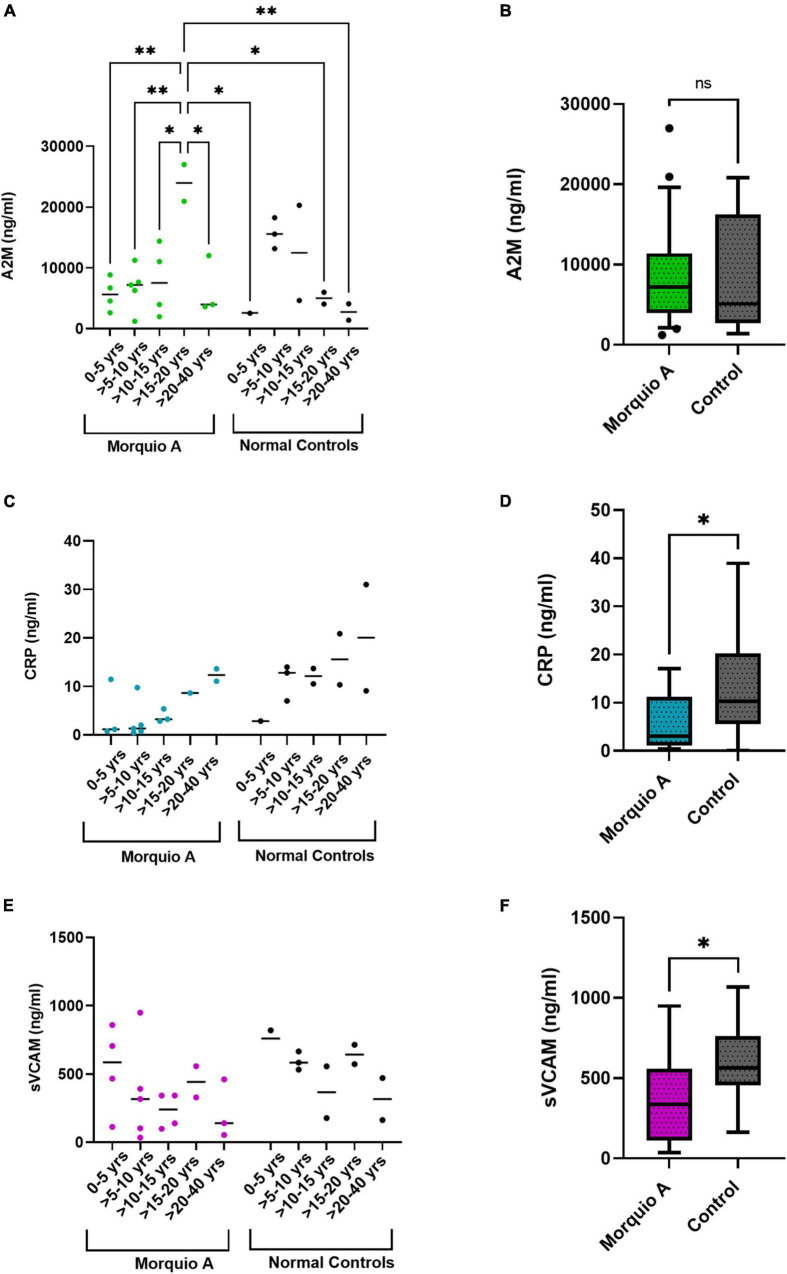
Comparison of cardiovascular biomarkers between Morquio A and normal controls. Scatter plot and comparison of **(A,B)** α-2-macroglobulin [A2M], **(C,D)** C-reactive protein [CRP], and **(E,F)** circulating vascular cell adhesion molecule-1 [sVCAM-1], between Morquio A and normal controls stratified **(A,C,E)** or non-stratified **(B,D,F)** by age groups. Significant differences are denoted with asterisks (*p ≤ 0.05, ^**^p ≤ 0.01). *p*-values were determined by one-way ANOVA followed by ordinary one-way ANOVA multiple comparisons test.

To investigate whether changes in ECM biomarkers were related to the severity of CVD in Morquio A patients, we looked at three different CVD biomarkers in a subset of samples (Table 5). We measured levels of α-2-macroglubulin (A2M) and C-reactive protein (CRP) which are associated with chronic inflammation causing plaque development ending with clinical ischemic complications. We found that although there was difference in A2M levels at different ages in Morquio A and normal controls (*p* = 0.0034) (Figure 6A), there was no significant difference overall between those two populations (Figure 6B). In contrast, levels of CRP increased with age in both Morquio A and normal controls (Figure 6C), and the overall levels of CRP were significantly higher in normal controls (*p* = 0.018) when compared to Morquio A patients (Figure 6D). We also explored levels of circulating vascular cell adhesion molecule-1 (sVCAM-1) in Morquio A patients, which is indicative of endothelial dysfunction. We found that the sVCAM-1 levels are higher in Morquio A patients between 0 and 5 years of age (Figure 6E) and the overall levels in Morquio A patients were significantly lower than those in the normal controls (Figure 6F).

**TABLE 5:** Demographic and cardiovascular disease biomarker comparisons of Morquio A patients and normal controls.

	Morquio A	Normal controls	Morquio A vs. Control
	(*n* = 22)	(*n* = 16)	
	Median (IQR); n	Median (IQR); n	*P*-value
**Demographics**			
Age (years)	9.5 (1.8–18.4); 22	11 (6–18);16	0.571
**Biomarkers**			
Cathepsin S (ng/mL)	7.17 (6.06–11.45); 22	12.55 (10.05–18.50); 16	0.001
Elastin (ng/ml)	3.09 (2.105–4.116); 21	2.832 (2.280–4.045); 14	0.795
Plasma KS (ng/mL)	394.0 (221.6–603.5); 16	–	–
Urine GAGs (mg/gCr)	168.9 (88.60–352.5); 18	–	–
Urine KS (mg/gCr)	4.29 (2.066–13.00); 18	–	–
Plasma GAGs (ng/mL)	–	–	–
A2M (ng/ml)	6945 (3965–20949); 20	5086 (2679–16249); 16	0.730
CRP (ng/ml)	3.21 (1.21–11.34); 17	10.30 (5.551–20.21); 15	0.019
sVCAM-1 (ng/ml)	335.3 (110.5–556.7); 22	564.3 (454.1–760.9); 16	0.018

*p-value; Welch’s test.*

**FIGURE 7:**
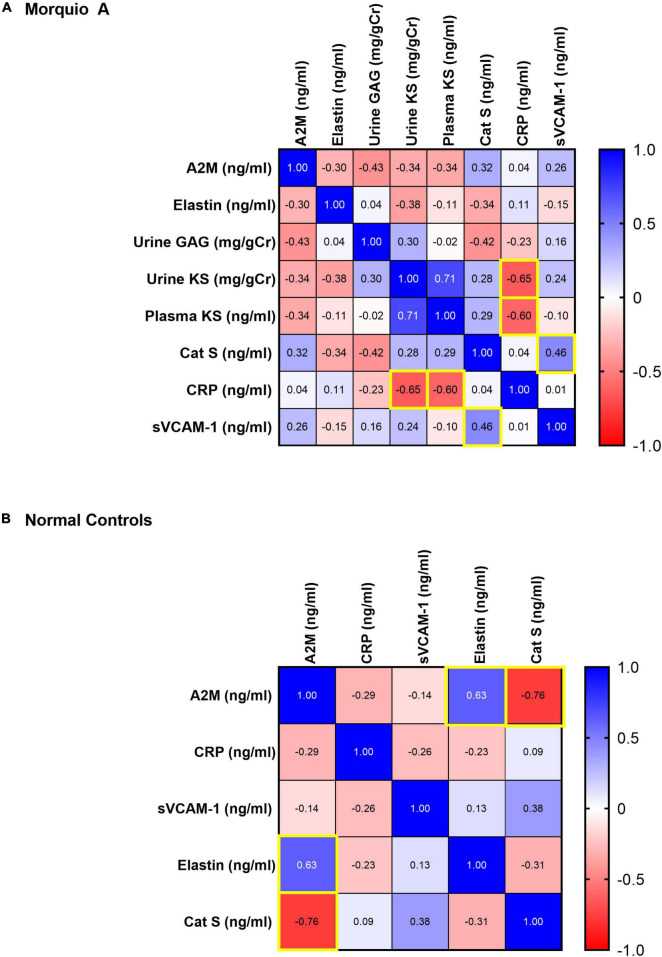
Pairwise Pearson correlation matrix of **(A)** α-2-macroglobulin (A2M), C-reactive protein (CRP), circulating vascular cell adhesion molecule-1 (sVCAM-1), elastin, cathepsin S, keratan sulfate (KS), and glycosaminoglycans (GAGs) in Morquio A samples, and **(B)** A2M, CRP, sVCAM-1, elastin, and cathepsin S in normal controls. Blue indicates positive correlation, and red indicates negative correlation. Darker colors are associated with stronger correlation coefficients. Yellow boxes indicate significant correlations.

To further investigate the relationship of these three CVD biomarkers and the ECM biomarkers, we measured their correlation. A moderate to strong inverse correlation existed among all age groups for urine KS and CRP (*p* = 0.013) as well as plasma KS and CRP (*p* = 0.022) in Morquio A patients. In addition, we found a moderate correlation between sVCAM-1 and Cathepsin S in Morquio A patients at all ages (*p* = 0.03) (Figure 7A). In normal controls, we found a strong inverse correlation between Cathepsin S and A2M (*p* = 0.001) and a moderate to strong correlation between elastin and A2M (*p* = 0.01) (Figure 7B).

## Discussion

Cardiovascular disease in Morquio A remains the second leading cause of death. It has been documented as early as infancy and it progresses with age. Current therapeutic options have shown limited to no impact on CVD progression. This study aimed to discover novel lifesaving biomarkers for the diagnosis, prognosis, and treatment of CVD in this patient population.

This study is the first to investigate cathepsin S and elastin levels as biomarkers to assess the severity of CVD, genotype, and phenotype in Morquio A Syndrome. We found that cathepsin S levels in Morquio A patients were significantly lower when compared to normal controls independent of patient’s severity. This reflects that cathepsin S activity may be impeded in Morquio A. Cathepsin S must be activated and secreted from the lysosome into the ECM to cleave its substrates and its remodeling (50). It has been previously shown that large amounts of GAGs, in particular C4S and C6S, within the lysosome can alter and affect the maturation of cathepsin S (51). Stability and enhanced activation of cathepsin S into its functioning form occurs in the presence of physiologic levels of GAGs (50). This may explain our findings of increased cathepsin S levels in normal controls, especially in the young pediatric age range. GAG levels in pediatric controls are the highest in early childhood and decrease with cessation of growth into adulthood (45, 46, 52). Our experiments displayed similar findings in our pediatric normal control population. Cathepsin S activity may be enhanced by the increased presence of GAGs in early normal control childhood resulting in higher cathepsin S levels during this time frame. This contrasts to young Morquio A children who tend to display large, excessive amounts of GAGs during childhood which may be altering cathepsin S activity with subsequent reflection in decreased levels.

Furthermore, our findings suggest that cathepsin S levels can be used to discriminate Morquio A children from normal controls of all ages, especially early childhood. To date, there is no published reference range for circulating cathepsin S in human plasma. Previous studies looking at cathepsin S in various human disease processes report serum and/or plasma levels in age matched healthy controls >20 (53–58). Cathepsin S levels in the pediatric age range have yet to be reported. Our study is the first to investigate cathepsin S levels in the pediatric population and in Morquio A disease. To the best of our knowledge, there exists no study on cathepsin S in serum and plasma of MPS patients. We note the existence of cathepsin studies in animal models of MPS I (59), II (60), III (59), or VII (28, 31, 61).

Cathepsin S has been associated with the pathogenesis of many conditions including but not limited to lung diseases (62), autoimmune diseases (56, 57, 63, 64), CVD (36, 65), type 2 diabetes (66, 67), obesity (58, 68), metabolic syndrome (55), neurodegenerative disease (69), and cancer (70, 71). The majority of studies report elevated cathepsin S levels in association with the specific disease process, however, a recent study has reported decreased cathepsin S levels in patients with systemic sclerosis associated interstitial lung disease (72).

In normal controls, we discovered that elastin increased throughout childhood then decreased until middle adulthood before elevating again. These findings may be explained by normal elastin fiber formation. Elastogenesis is a complex hierarchal process compromised of many distinct phases that begins with tropoelasin synthesis. Tropoelastin synthesis mainly occurs perinatally with elastin fiber formation and deposition occurring prior to birth and throughout childhood (73). *De novo* elastin synthesis normally terminates leading up to and following adolescence (32). While the majority of tropoelastin synthesis occurs perinatally, synthesis has also been shown to occur in response to tissue damage (73). Tissue damage occurs with the normal aging process (74). Our findings of increased elastin levels after age 40 could be explained by increasing tropoelastin synthesis in response to the normal tissue damage that occurs with aging.

In contrast to the normal control findings, elastin levels in Morquio A patients tended to increase with age with highest levels after 40 years of age. This too may be explained by the presence of large amounts of GAGs in Morquio A childhood. Elastogenesis or the formation of elastic fibers occurs very early in life and disappears by the onset of puberty (75). The presence of high amounts of GAGs in the ECM in early Morquio A childhood could prevent the proper assembly of elastic fibers or cause irreversible damage. For example, it has been speculated that marked accumulated GAGs in arteries cause swelling and separation of elastic lamella (76). As GAG levels decrease into adolescence and adulthood, the remaining elastic fibers may assemble properly reflecting the increase in elastin levels. Cell culture studies have shown that GAGs induce production of tropoelastin in cells and upregulate microfibrillar associated genes leading to the formation of elastic fibers (77). Thus, the presence of accumulated GAGs in early Morquio childhood could be leading to induction of tropoelastin, a soluble precursor of elastin essential to elastin fiber assembly. Tropoelastin then must be chaperoned outside of the cell to form elastin in the ECM (73). These same intracellular GAGs that induce tropoleastin production may affect the actual elastin fiber synthesis pathway. This has been shown *in vitro* in MPS I in which increased dermatan sulfate leads to a deficiency of elastic binding protein which is a key chaperone for elastin binding synthesis (78). As GAGs decrease throughout childhood into adulthood, proper elastin fiber assembly can be achieved from the remaining intracellular tropoelastin which would explain gradual increase in elastin levels observed in our study.

Our study suggests that elastin levels can be used to discriminate adolescent and adult Morquio patients from normal controls. Like cathepsin S, there is no published reference ranges for circulating plasma and/or serum elastin. Previous studies have mainly explored levels of elastin derived peptides or anti-elastin antibodies in pathological disease processes (79–83). The relationship of elastin protein levels to elastin derived peptides and anti-elastin antibodies has yet to be explored. Our study is the first to explore plasma elastin protein levels in the normal population and in a pathological disease. In addition, our findings confirm that urinary KS is associated with clinical severity of Morquio A (52, 84).

Our experiments show that sVCAM-1 levels in Morquio A patients are lower than in normal controls, which can be explained by the dysregulation of the vascular endothelial glycocalyx in MPS patients. In CVD, inflammatory activation of endothelial cells triggers the expression of some leukocyte adhesion molecules including sVCAM-1 followed by the migration of monocytes in the tunica intima of the arterial wall thus amplifying the inflammatory response (85, 86). Chondroitin sulfate (CS) represents the largest population of arterial GAGs, therefore it actively participates in the development of arterial disease (87). Patients with Morquio A disease showed increased cIMT (16) and their endothelial dysfunction/dysregulation may be primed by the accumulation of C6S developing atherosclerotic lesions. Since CS proteoglycans play a key role in the organization and assembly of ECM, the continuous accumulation of CS and KS substrate cause widespread dysregulation including inhibition of elastic fiber assembly and cell adhesion molecules (88).

C-reactive protein (CRP) and α2M are biomarkers widely used to predict severity of CVD. CRP is known to modulate innate immune response, promote platelet activation, vascular remodeling, and angiogenesis (89). Recent studies showed that CS intake is associated with a reduction in CRP concentration in blood, indicating decrease in inflammation (90–92). These findings, along with the significantly lower CRP levels in Morquio A patients found in this study, open a new direction in this field to gain insights in the understanding of vascular endothelial glycocalyx dysregulation in this patient population.

Identification of life-saving novel biomarkers remains the main therapeutic target in Morquio A CVD. This novel study is the first to explore the relationship of cathepsin S and elastin in Morquio A and the normal population. We discovered that cathepsin S has promising attributes as a biomarker in Morquio A children. Elastin, on the other hand, has promising attributes as a biomarker in Morquio A adolescents and adults. Further studies are needed to understand how cathepsin S and elastin correlate with Morquio A severity and treatment outcomes.

## Limitations

Our study also had several limitations. First, our study was underpowered in several age groups which may have impacted results of age group dependent analyses. Second, urine was not collected from the normal pediatric patients limiting comparisons of urinary GAGs and urinary KS in age dependent analyses. Similarly, plasma GAGs were not analyzed on Morquio A patients or normal adults further limiting comparisons. Importantly, patients’ database was de-identified so we could not reach out to patients to obtain echocardiograms or ultrasounds to have additional clinical information on the CVD status in patients without reported known cardiovascular symptoms. Lastly, we were unable to compare cathepsin S and elastin levels found in this study to previous studies as this is the first study to explore pediatric and adult aged Morquio A patients and healthy normal controls.

## Data Availability Statement

The original contributions presented in the study are included in the article/Supplementary Material, further inquiries can be directed to the corresponding author.

## Ethics Statement

The studies involving human participants were reviewed and approved by the Institutional Review Board (IRB) at Saint Louis University. Written informed consent for participation was not required for this study in accordance with the national legislation and the institutional requirements.

## Author Contributions

AM conceived and supervised the study. BM and AM designed the study and wrote the manuscript. BM, LW, and QG performed laboratory analysis. AA contributed to the analysis of CVD biomarkers. BM, AA, and AM performed statistical analysis. BM, LW, QG, AA, and AM critically reviewed the manuscript. All authors contributed to the article and approved the submitted version.

## Conflict of Interest

The authors declare that the research was conducted in the absence of any commercial or financial relationships that could be construed as a potential conflict of interest.

## Publisher’s Note

All claims expressed in this article are solely those of the authors and do not necessarily represent those of their affiliated organizations, or those of the publisher, the editors and the reviewers. Any product that may be evaluated in this article, or claim that may be made by its manufacturer, is not guaranteed or endorsed by the publisher.
