# Supplementary material for: Mucopolysaccharidosis Type IVA: Extracellular Matrix Biomarkers in Cardiovascular Disease
Source: Front Cardiovasc Med. 2022 May 10;9:829111. doi: 10.3389/fcvm.2022.829111 (PMC9127057; doi:10.3389/fcvm.2022.829111)
Supplement: Supplementary file 1 [file Table_1.pdf]

Supplemental Table 1: Molecular characteristics of patients with Morquio A disease who participated in study

Patient	Phenotype	Gender	Age (years)	Allele 1				Allele 2				GALNS activity (nmol/mg) NR:1.4-7.5
				Genotype	Genotype severity	*In-silico* Mutation score	Damaging*	Genotype	Genotype severity	*In-silico* Mutation score	Damaging*	
1	s	F	1.8	p.G301C	severe	1.00	probably	p.G301C	severe	1.00	probably	0
2	s	M	1.9	p.R386C	severe	1.00	probably	p.R386C	severe	1.00	probably	0.23
3	m	F	2.0	N.A.	N.A.	N.A.	N.A.	N.A.	N.A.	N.A.	N.A.	0
4	s	M	2.7	p.D233N	severe	1.00	probably	N.A.	N.A.	N.A.	N.A.	0
5	s	F	3.5	p.G247D	severe	0.75	probably	p.G301C	severe	1.00	probably	0
6	m	M	4.0	N.A.	N.A.	N.A.	N.A.	N.A.	N.A.	N.A.	N.A.	0
7	s	M	4.0	N.A.	N.A.	N.A.	N.A.	N.A.	N.A.	N.A.	N.A.	0
8	s	M	4.3	p.M41RfsX46	severe	1.00	probably	p.I113F	severe	0.99	probably	0
9	s	F	4.4	p.L345P	severe	1.00	probably	p.D233N	severe	1.00	probably	0.26
10	s	F	4.5	p.G116S	severe	1.00	probably	p.R386C	severe	1.00	probably	0
11	m	M	5.5	p.M391V	attenuated	0.56	possibly	p.M391V	attenuated	0.56	possibly	N.A.
12	s	M	5.5	p.M41L	severe	0.98	probably	p.M41L	severe	0.98	probably	0.49
13	m	M	6.0	p.A107GfxX54	severe	1.00	probably	N.A.	N.A.	N.A.	N.A.	0.62
14	s	F	6.0	⊿F284/258+W10X	severe	1.00	probably	p.W10X	severe	1.00	probably	0
15	s	M	6.0	p.L307P	severe	1.00	probably	p.S341R	severe	0.96	possibly	N.A.
16	s	F	6.3	N.A.	N.A.	N.A.	N.A.	N.A.	N.A.	N.A.	N.A.	0
17	s	F	6.4	IVS8-2A→G	severe	1.00	probably	N.A.	N.A.	N.A.	N.A.	0.15
18	s	M	6.5	N.A.	N.A.	N.A.	N.A.	N.A.	N.A.	N.A.	N.A.	0
19	s	F	6.9	p.P77R	severe	1.00	probably	p.P77R	severe	1.00	probably	N.A.
20	s	F	6.9	p.S162F	severe	1.00	probably	p.S162F	severe	1.00	probably	0
21	m	M	7.0	p.R380S	attenuated	0.99	probably	1356 insT	mild	NA	NA	N.A.

22	s	F	7.0	N.A.	N.A.	N.A.	N.A.	N.A.	N.A.	N.A.	N.A.	0
23	m	F	7.2	p.M391V	attenuated	0.56	probably	p.F452I	attenuated	0.43	benign	0.67
24	m	M	7.2	p.W10X	severe	1.00	probably	N.A.	N.A.	N.A.	N.A.	0.75
25	s	M	8.0	p.P77R	severe	1.00	probably	p.P77R	severe	1.00	probably	0.28
26	s	M	8.2	p.G139S	severe	1.00	probably	p.G301C	severe	1.00	probably	0
27	m	F	8.5	p.M391V	attenuated	0.56	possibly	p.M391V	attenuated	0.56	possibly	N.A.
28	s	M	8.5	N.A.	N.A.	N.A.	N.A.	N.A.	N.A.	N.A.	N.A.	0
29	m	M	8.8	p.C308R	severe	1.00	probably	p.R253Q	attenuated	1.00	probably	N.A.
30	s	M	8.8	N.A.	N.A.	N.A.	N.A.	N.A.	N.A.	N.A.	N.A.	0
31	m	M	9.0	N.A.	N.A.	N.A.	N.A.	N.A.	N.A.	N.A.	N.A.	0
32	s	F	9.0	p.Q338X	severe	1.00	probably	p.M391V	attenuated	0.56	possibly	N.A.
33	s	F	10.0	p.G155E	severe	1.00	probably	p.L369P	severe	0.98	probably	N.A.
34	m	F	10.1	p.F284V	attenuated	0.30	benign	p.F284V	attenuated	0.30	benign	0
35	m	M	10.3	p.N495K	undef	1.00	probably	p.F452 I	attenuated	0.43	benign	0
36	s	M	10.4	p.L86P	severe	1.00	probably	p.M1I	severe	0.38	benign	0
37	m	M	10.7	N.A.	N.A.	N.A.	N.A.	N.A.	N.A.	N.A.	N.A.	0
38	m	M	11.0	p.M391V	attenuated	0.56	possibly	p.F452I	attenuated	0.43	benign	0.36
39	s	F	11.0	p.P77R	severe	1.00	probably	p.P77R	severe	1.00	probably	0.40
40	s	M	12.0	p.A291T	severe	1.00	probably	IVS 7+1 G-C	severe	1.00	probably	N.A.
41	s	N.A.	13.0	p.G301C	severe	1.00	probably	p.M391V	attenuated	0.56	possibly	N.A.
42	s	F	13.0	IVS 8+1 G → A	severe	1.00	probably	IVS 1-1 G → C	severe	1.00	probably	N.A.
43	m	M	14.0	p.Q111X	severe	1.00	probably	p.M391V	attenuated	0.56	possibly	N.A.
44	s	M	18.2	p.G42E	severe	1.00	probably	p.P125L	severe	1.00	probably	0
45	s	M	19.0	N.A.	N.A.	N.A.	N.A.	N.A.	N.A.	N.A.	N.A.	0.30
46	s	F	23.3	p.X523EextX93	severe	1.00	probably	p.M318R	severe	0.98	probably	0
47	m	M	24.0	p.M41L	severe	0.98	probably	p.M41L	severe	0.98	probably	N.A.

48	s	F	24.0	p.G47R	severe	1.00	probably	p.G47R	severe	1.00	probably	0
49	s	F	27.0	p.A75G	severe	0.71	possibly	A75G	severe	0.71	possibly	N.A.
50	m	M	38.0	p.R451S	attenuated	0.92	probably	p.R451S	attenuated	0.92	probably	0.06
51	s	F	45.5	N.A.	N.A.	N.A.	N.A.	N.A.	N.A.	N.A.	N.A.	0.40
52	m	N.A.	46.8	p.G247D	severe	0.75	possibly	p.Y254C	undefined	1.00	probably	0
53	m	F	47.0	p.W325C	attenuated	1.00	probably	N.A.	N.A.	N.A.	N.A.	0
54	s	N.A.	57.2	N.A.	N.A.	N.A.	N.A.	N.A.	N.A.	N.A.	N.A.	0

Abbreviations: m: mild; s: severe; N.A.: Not available

*Based on classification of Poly Phen2
